# Associations Between COVID-19 Misinformation Exposure and Belief With COVID-19 Knowledge and Preventive Behaviors: Cross-Sectional Online Study

**DOI:** 10.2196/22205

**Published:** 2020-11-13

**Authors:** Jung Jae Lee, Kyung-Ah Kang, Man Ping Wang, Sheng Zhi Zhao, Janet Yuen Ha Wong, Siobhan O'Connor, Sook Ching Yang, Sunhwa Shin

**Affiliations:** 1 School of Nursing University of Hong Kong Hong Kong China (Hong Kong); 2 College of Nursing Sahmyook University Seoul Republic of Korea; 3 School of Health in Social Science The University of Edinburgh Edinburgh United Kingdom; 4 School of Medicine, Medical Sciences and Nutrition University of Aberdeen Aberdeen United Kingdom

**Keywords:** COVID-19, misinformation, infodemic, infodemiology, anxiety, depression, PTSD, knowledge, preventive behaviors, prevention, behavior

## Abstract

**Background:**

Online misinformation proliferation during the COVID-19 pandemic has become a major public health concern.

**Objective:**

We aimed to assess the prevalence of COVID-19 misinformation exposure and beliefs, associated factors including psychological distress with misinformation exposure, and the associations between COVID-19 knowledge and number of preventive behaviors.

**Methods:**

A cross-sectional online survey was conducted with 1049 South Korean adults in April 2020. Respondents were asked about receiving COVID-19 misinformation using 12 items identified by the World Health Organization. Logistic regression was used to compute adjusted odds ratios (aORs) for the association of receiving misinformation with sociodemographic characteristics, source of information, COVID-19 misinformation belief, and psychological distress, as well as the associations of COVID-19 misinformation belief with COVID-19 knowledge and the number of COVID-19 preventive behaviors among those who received the misinformation. All data were weighted according to the Korea census data in 2018.

**Results:**

Overall, 67.78% (n=711) of respondents reported exposure to at least one COVID-19 misinformation item. Misinformation exposure was associated with younger age, higher education levels, and lower income. Sources of information associated with misinformation exposure were social networking services (aOR 1.67, 95% CI 1.20-2.32) and instant messaging (aOR 1.79, 1.27-2.51). Misinformation exposure was also associated with psychological distress including anxiety (aOR 1.80, 1.24-2.61), depressive (aOR 1.47, 1.09-2.00), and posttraumatic stress disorder symptoms (aOR 1.97, 1.42-2.73), as well as misinformation belief (aOR 7.33, 5.17-10.38). Misinformation belief was associated with poorer COVID-19 knowledge (high: aOR 0.62, 0.45-0.84) and fewer preventive behaviors (≥7 behaviors: aOR 0.54, 0.39-0.74).

**Conclusions:**

COVID-19 misinformation exposure was associated with misinformation belief, while misinformation belief was associated with fewer preventive behaviors. Given the potential of misinformation to undermine global efforts in COVID-19 disease control, up-to-date public health strategies are required to counter the proliferation of misinformation.

## Introduction

### Background

COVID-19 has brought significant challenges to public health with its high infectivity and severity, particularly in vulnerable groups (eg, older adults, those with chronic diseases) [1-4], leading to a rapid rise in cases worldwide. On top of managing the response to the COVID-19 global health crisis, the World Health Organization (WHO) and governments also face the challenge of an “infodemic,” which causes people to experience difficulties in finding credible and trustworthy sources amid an excess of information [5]. Although the concept of an infodemic is not a new one, the digital age we are currently living in has magnified its effects and added complexities to the challenge it poses. With the widespread use of social media such as internet websites, social networking services (SNSs), and instant messaging services, people all over the world are more connected than ever, allowing information to be shared easily and quickly [6]. However, a recent study has found that in the midst of the global pandemic, COVID-19 misinformation is just as likely to spread and engage users on social media platforms as accurate information, which can pose an equal threat to the COVID-19 public health response by affecting public awareness and knowledge of the disease [7].

Misinformation can be defined as information that is false or inaccurate and not supported by scientific evidence [8]. Misinformation in the context of COVID-19 can include inaccurate information regarding the virus and its transmission, conspiracy theories, and fabricated reports regarding methods of prevention and treatment [9]. Some of its consequences include the panic-buying and hoarding of goods, taking ineffective and potentially harmful remedies, ignoring advice from health authorities, and engaging in behavior that increases the risk of virus transmission [10]. Despite the many efforts by the WHO and public health organizations to battle the infodemic, such as conducting campaigns against COVID-19 misinformation, cooperating with social media platforms, and regularly providing evidence-based information to the public (eg, COVID-19 advice for the public: myth busters [11]), the proliferation of misinformation worldwide has remained rampant [9,12,13]. Although social media can be used effectively to provide essential health-related information to the global community, misinformation does not require professional verification or review, and thus has the potential to proliferate quicker and be disseminated farther on social media due to existing algorithms that highlight popular or desired content. This highlights the tall challenge health authorities face in delivering accurate information to the public in precedence to the proliferation of misinformation [4,5] and the need for new strategies to build preparedness [9] against future infodemics.

South Korea (hereafter Korea) has one of the most developed technological infrastructures in the world, and the use of the internet is well-integrated into Koreans’ everyday lives. As such, information and communication technology (ICT) has also been employed in Korea’s response to the COVID-19 pandemic, including the monitoring and tracking of COVID-19 cases, conveying of health information to professionals and the public, and for allocating and distributing resources, such as COVID-19 test kits and protective equipment [14,15]. With the deep integration of ICT such as social media into Koreans’ daily lives and activities [6], it is expected that Koreans will be highly exposed to COVID-19 information and misinformation, through either active searches for information or passive receiving of information through messages, emails, or news feeds. Notwithstanding our awareness of misinformation and the risk it poses to public health, there remains little evidence on the prevalence of COVID-19 misinformation exposure and its effects on health beliefs and behaviors, including psychological well-being [16].

### Objectives

We aimed to investigate the following: (1) the prevalence of COVID-19 misinformation exposure and misinformation belief, (2) associated factors including psychological distress with misinformation exposure; and (3) the associations between misinformation exposure and COVID-19 knowledge as well as preventive behaviors in Korean adults.

## Methods

### Study Design and Sampling

A cross-sectional online survey was conducted using the largest online survey platform in Korea [17]. This platform was chosen as it has 5 million survey panel members nationwide (as of 2020) and has been used to conduct more than 160,000 surveys for academic (such as those by Kim et al [18] and Ra et al [19]), government, and industry research. The inclusion criteria were the following: (1) aged ≥20 years (according to Korea’s civil law, those aged ≥20 years are regarded as adults), (2) a resident in the Seoul Metropolitan area (including the Seoul, Gyeonggi-do, and Incheon areas, in which 50.0% of the Korean population resides as of 2020), (3) has encountered COVID-19 information from any source, and (4) a Korean speaker.

The company sent survey invitations containing general information about the survey such as its aim and participation incentive (KRW 1000 [US $1 is about KRW 1200]) via emails and a smartphone app to registered survey panel members who met the inclusion criteria on April 23, 2020. The survey closed on the same date (ie, recruitment was conducted for one day due to the cost involved). Details of the survey and consent statement were provided on the first page of the online survey. Respondents provided consent by clicking “Agree to participate in this survey” on the same page, before moving on to answer the survey questions. The survey took approximately 15 minutes to complete. The survey questionnaire is attached as Multimedia Appendix 1. Ethical approval was obtained from an institutional review board at Sahmyook University in Seoul (Ref: 2-1040781-A-N-012020021HR). Meanwhile, as of April 23, 2020, Korea had 10,708 confirmed cases of COVID-19 since the first case was reported on January 24, 2020. The daily new cases of COVID-19 peaked on March 3, 2020 (803 cases), after which there was a downward trend until April 23, 2020 (14 new cases).

### Measurements

Sociodemographic characteristics including sex, age, education level, household arrangement, and monthly personal income were collected.

COVID-19 misinformation items used in this study were extracted from COVID-19 misinformation reports by the WHO [11,20], the main coordinator of the global COVID-19 pandemic response. In total, 12 misinformation items about COVID-19 transmission, infectivity, prevention, and treatment were included (see Question 2 in Multimedia Appendix 1). Respondents were allocated to one of two groups: misinformation exposure (defined as having seen one or more items of misinformation, through active searching or passive receiving means) or misinformation nonexposure. Respondents were then asked if they believed any of the 12 misinformation items to be correct (hereafter misinformation belief) or incorrect.

Measures for psychological distress included anxiety and depressive symptoms, using the Patient Health Questionnaire-4 (PHQ-4; four items), which consists of two subscales: the Generalized Anxiety Disorder-2 (GAD-2; two items) and Patient Health Questionnaire-2 (PHQ-2; two items) [21]. The score of each subscale ranges from 0 to 6, and a score of ≥3 indicates a high risk of anxiety (GAD-2) and depression (PHQ-2), respectively [21]. PHQ-4 was validated in Korean (Cronbach α=.79; acceptable convergent validity) [22]. An additional Primary Care Post-Traumatic Stress Disorder Screen for DSM-5 (PC-PTSD-5; five items) that was validated in Korean (Cronbach α=.87; acceptable concurrent validity) [23,24] was also adopted to screen respondents for posttraumatic stress disorder (PTSD) risk. The scores range from 0 to 5 and the cutoff score for high risk of PTSD is 3.

COVID-19 knowledge was assessed using five COVID-19 knowledge questions (definition, transmission modes, main symptoms, prevention, and treatment) that were extracted from a questionnaire developed by the WHO [25] (see Questions 4-8 in Multimedia Appendix 1). Higher scores indicate a higher COVID-19 knowledge level.

The number of COVID-19 preventive behaviors that the respondents performed during the past three months was assessed with 10 answer options that were extracted from the COVID-19 preventive methods recommended by the WHO [25] and Korea Centers for Disease Control and Prevention [26], such as washing hands regularly, covering one’s mouth and nose when coughing, and social distancing (see Question 9 in Multimedia Appendix 1). Higher scores indicated a higher engagement in COVID-19 preventive behaviors.

### Statistical Analysis

All data were weighted by age and sex distributions in the Seoul Metropolitan area, according to the Korea census data in 2018 [27]. Descriptive statistics were reported in numbers, proportions, means, and standard deviations (SD), as appropriate. The differences between the two groups (COVID-19 misinformation exposure group versus nonexposure group), including the respondents’ sociodemographic characteristics, source of information, COVID-19 misinformation belief, psychological well-being (ie, anxiety, depressive, and PTSD symptoms), and COVID-19 knowledge and preventive behaviors were analyzed by chi-square test or *t* test, as appropriate. The responses on the knowledge and preventive behavior questions were categorized into binary groups according to the mean scores for the chi-square analysis. Logistic regression was used to compute odds ratios (ORs) and adjusted ORs (aORs) to identify the association of misinformation exposure (a binary variable) with the sociodemographic characteristics, source of information, and COVID-19 misinformation belief. The associations of COVID-19 misinformation belief with COVID-19 knowledge and COVID-19 preventive behaviors among the respondents who encountered misinformation were also investigated. As subgroup analyses, we included the interaction term to test if demographic characteristics modify the associations of COVID-19 knowledge, preventive behaviors, misinformation belief, and psychosocial distress following misinformation exposure. Sex, age, highest education level, household arrangement, and monthly personal income were adjusted for the adjusted regression models. STATA 15 (StataCorp LLC) was used to conduct all analyses.

## Results

Of 1054 people who initially responded to the survey, five were excluded from the study as they reported that they had not encountered any COVID-19 information and therefore could not complete the survey. Among the 1049 respondents, 50.04% (n=505) were male, the mean age was 40.60 years (SD 12.87), 74.94% (n=786) had tertiary education or higher, 88.50% (n=929) lived with others, and 55.95% (n=587) had a monthly personal income <KRW 3,000,000 (US $2500; the average monthly income among those employed was KRW 2,970,000 [US $2475] in 2018; Table 1).

**Table 1:** Characteristics of COVID-19 misinformation exposure by respondents’ demographics, and sociobehavioral and psychological symptoms (N=1049).

Variables		Participants, n (%)^a^	Weighted values^b^, n (%)^a^	Misinformation		*P* value^c,d^
		N=1049	N=1049	Not exposed (n=338)	Exposed (n=711)	
**Sex**						
	Male	446 (42.52)	525 (50.04)	179 (53.08)	346 (48.60)	.15^c^
	Female	603 (57.48)	524 (49.96)	158 (46.92)	366 (51.40)	.15^c^
**Age (years), mean (SD)**		39.51 (10.47)	43.60 (12.87)	45.86 (13.01)	42.53 (12.67)	.02^d^
	20-29	198 (18.88)	196 (18.69)	48 (14.09)	149 (20.87)	.06^c^
	30-39	351 (33.46)	219 (20.85)	65 (19.25)	154 (21.61)	.06^c^
	40-49	311 (29.65)	243 (23.17)	78 (23.14)	165 (23.18)	.06^c^
	50-59	148 (14.12)	236 (22.54)	74 (22.11)	162 (22.74)	.06^c^
	60-69	41 (3.91)	154 (14.75)	72 (21.41)	82 (11.59)	.06^c^
**Education**						
	High school or below	233 (22.21)	263 (25.06)	92 (27.13)	171 (24.08)	.83^c^
	Tertiary or above	816 (77.79)	786 (74.94)	246 (72.87)	540 (75.92)	.83^c^
**Household arrangement**						
	Living alone	142 (13.54)	121 (11.50)	32 (9.55)	88 (12.43)	.10^c^
	Living with others	907 (86.46)	929 (88.50)	306 (90.45)	623 (87.57)	.10^c^
**Monthly personal income, KRW** **(US $)**						
	<3,000,000 (<2500)^e^	622 (59.29)	587 (55.95)	165 (48.78)	422 (59.35)	.03^c^
	3,000,000-4,990,000 (2500-4158)	264 (25.17)	261 (24.92)	99 (29.24)	163 (22.86)	.03^c^
	≥5,000,000 (>4158)	163 (15.54)	201 (19.14)	74 (21.98)	127 (17.79)	.03^c^
**COVID-19 information source^f^**						
	Television, radio, or newspaper (offline)	999 (95.23)	998 (95.07)	316 (93.53)	682 (95.80)	.40^c^
	Television, radio, or newspaper (online)	1036 (98.76)	1030 (98.21)	328 (97.06)	703 (98.76)	.54^c^
	Other internet websites	864 (82.36)	842 (80.26)	262 (77.55)	580 (81.55)	.44^c^
	Social network services	753 (71.78)	754 (71.82)	220 (65.10)	534 (75.01)	.03^c^
	Instant messaging	873 (83.22)	868 (82.71)	262 (77.49)	606 (85.19)	.011^c^
**COVID-19 misinformation belief**						
	No	604 (57.58)	618 (58.86)	291 (86.21)	326 (45.87)	<.001^c^
	Yes	445 (42.42)	432 (41.14)	47 (13.79)	385 (54.13)	<.001^c^
**Anxiety symptom, mean (SD)^g^**		1.49 (1.60)	1.51 (1.65)	3.18 (1.58)	3.66 (1.66)	<.001^d^
	No	853 (81.32)	854 (81.38)	290 (85.94)	564 (79.22)	.002^c^
	Yes	196 (18.68)	195 (18.62)	48 (14.06)	148 (20.78)	.002^c^
**Depressive symptom, mean (SD)^h^**		2.02 (1.73)	2.04 (1.78)	3.76 (1.78)	4.17 (1.77)	.001^d^
	No	738 (70.35)	739 (70.45)	253 (74.85)	486 (68.36)	.01^c^
	Yes	311 (29.65)	310 (29.55)	85 (25.15)	225 (31.64)	.01^c^
**Posttraumatic stress disorder symptom, mean (SD)^i^**		1.43 (1.09)	1.43 (1.14)	1.12 (1.04)	1.57 (1.15)	<.001^d^
	No	902 (85.99)	903 (86.05)	306 (90.52)	597 (83.92)	.002^c^
	Yes	147 (14.01)	146 (13.95)	32 (9.48)	114 (16.08)	.002^c^
**COVID-19 knowledge, mean (SD)**		24.69 (2.56)	24.65 (2.69)	24.79 (2.24)	24.58 (2.88)	.38^d^
	Low (0-24)	444 (42.33)	446 (42.55)	142 (41.97)	305 (42.82)	.99^c^
	High (25-35)	605 (57.67)	603 (57.45)	196 (58.03)	407 (57.18)	.99^c^
**COVID-19 preventive behaviors, mean (SD)**		6.94 (2.42)	7.01 (2.47)	7.01 (2.46)	7.02 (2.48)	.72^d^
	0-6 behaviors	396 (37.75)	388 (36.99)	129 (38.15)	259 (36.44)	.70^c^
	≥7 behaviors	653 (62.25)	661 (63.01)	209 (61.85)	452 (63.57)	.70^c^

^a^Calculated percentages were rounded off to one decimal place; accordingly, combined percentages can exceed 100%.

^b^Data were weighted by sex and age distribution of the general population in the Seoul metropolitan area.

^c^*P* for chi-square (computed using unweighted data).

^d^*P* for *t* test (computed using unweighted data).

^e^Average monthly income among employees was KRW 2,970,000 in 2018.

^f^Multiple responses allowed.

^g^Generalized Anxiety Disorder Questionnaire-2 (GAD-2) score ≥3.

^h^Patient Health Questionnaire-2 (PHQ-2) score ≥3.

^i^Primary Care Post-Traumatic Stress Disorder Screen for DSM-5 (PC-PTSD-5) score ≥3.

The majority of the respondents encountered COVID-19 information from diverse media including television, newspapers, internet websites, SNSs, and instant messaging. Overall, 57.45% (n=603) had high levels (score of 25-35) of COVID-19 knowledge and 63.01% (n=661) reported conducting ≥7 COVID-19 preventive behaviors. In total, 18.68% (n=196), 31.55% (n=331), and 13.95% (n=146) reported anxiety, depressive, and PTSD symptoms, respectively (Table 1). Overall, 41.14% (n=432) reported believing in one or more of the 12 COVID-19 misinformation items, while exposure to at least one COVID-19 misinformation item in the preceding three months was reported by 67.78% (n=711) of the respondents. In addition, 49.76% (n=354) and 48.14% (n=342) encountered misinformation about reusing masks (Table 2), which was the most common item of misinformation reported.

**Table 2:** Respondents’ exposure to COVID-19 misinformation (N=1049).

Variables		Participants, n (%)^a^	Weighted values^b^, n (%)^a^
**Number of COVID-19 misinformation items**			
	0	321 (30.60)	338 (32.19)
	1	279 (26.60)	260 (24.74)
	2	205 (19.54)	206 (19.63)
	≥3 items	244 (23.26)	246 (23.44)
**COVID-19 misinformation items^c^**		728 (69.40)	711 (67.78)
	Masks can be sterilized and reused after steaming with hot water	346 (47.53)	354 (49.76)
	Masks can be reused after spraying alcohol on its surface	345 (47.39)	342 (48.14)
	Drinking tea can prevent infection	231 (31.73)	219 (30.78)
	Gargling can disinfect the respiratory tract to prevent infection	150 (20.60)	168 (23.55)
	Coronavirus is artificially developed	172 (23.63)	156 (21.88)
	Basking in the sun can prevent infection	110 (15.11)	109 (15.30)
	Gargling with salt can prevent infection	104 (14.29)	109 (15.29)
	Taking antibiotics can prevent or treat infection	94 (12.91)	102 (14.34)
	Flip the sides of a used mask to reuse it	90 (12.36)	90 (12.60)
	Drinking alcohol/smoking can prevent infection	59 (8.10)	70 (9.88)
	Only older adults can be infected	25 (3.43)	32 (4.46)
	A vaccine is available now	15 (2.06)	14 (1.99)

^a^Calculated percentages were rounded off to one decimal place; accordingly, combined percentages can exceed 100%.

^b^Data were weighted by sex and age distribution of the general population in the Seoul metropolitan area in Korea.

^c^The COVID-19 misinformation items were extracted from World Health Organization documents [11,20].

COVID-19 misinformation exposure was negatively associated with being older (60-69 years age group: aOR 0.40, 95% CI 0.25-0.64 versus 20-29 years age group) and having a higher monthly personal income (KRW 3,000,000-4,990,000 [US $2500-$4158]: aOR 0.66, 95% CI 0.47-0.93 versus <KRW 3,000,000 [US $2500]). COVID-19 misinformation exposure was positively associated with a tertiary education or above (aOR 1.42, 95% CI 1.02-1.96 versus high school or below). Of the information sources, misinformation exposure was associated with SNSs (aOR 1.75, 95% CI 1.31-2.35 versus other information sources) and instant messaging (aOR 1.79, 95% CI 1.27-2.51 versus other information sources), while offline and online television, radio, and newspapers and other websites were not statistically significant. Misinformation exposure was also significantly associated with misinformation belief (aOR 7.33, 95% CI 5.17-10.38 versus no misinformation belief) and with psychological distress, including anxiety (aOR 1.80, 95% CI 1.24-2.61 versus no anxiety symptoms), depressive (aOR 1.47, 95% CI 1.09-2.00 versus no depressive symptoms), and PTSD symptoms (aOR 1.97, 95% CI 1.42-2.73 versus no PTSD symptoms). However, misinformation exposure was not associated with COVID-19 knowledge and preventive behaviors (Table 3).

**Table 3:** Associated factors with COVID-19 misinformation exposure (N=1049)^a^.

Variables		Misinformation exposure (yes/no)	
		Crude odds ratio (95% CI)	Adjusted odds ratio (95% CI)^b^
**Sex**			
	Male	Reference	Reference
	Female	1.20 (0.92-1.55)	1.15 (0.87-1.52)
**Age (years)**			
	20-29	Reference	Reference
	30-39	0.76 (0.49-1.17)	0.74 (0.46-1.17)
	40-49	0.68 (0.44-1.03)	0.70 (0.45-1.10)
	50-59	0.69 (0.45-1.06)	0.76 (0.48-1.20)
	60-69	0.37 (0.23-0.58)^c^	0.40 (0.25-0.64)^c^
**Education**			
	High school or below	Reference	Reference
	Tertiary or above	1.17 (0.87-1.58)	1.42 (1.02-1.96)^d^
**Household arrangement**			
	Living alone	Reference	Reference
	Living with others (including family)	0.74 (0.49-1.14)	0.83 (0.53-1.29)
**Monthly personal income, KRW (US $)^e^**			
	<3,000,000 (<2500)	Reference	Reference
	3,000,000-4,990,000 (2500-4158)	0.64 (0.47-0.87)^f^	0.66 (0.47-0.93)^d^
	≥5,000,000 (≥4158)	0.67 (0.47-0.93)^d^	0.74 (0.51-1.07)
**COVID-19 information source^g^**			
	Television, radio, or newspaper (offline)	1.58 (0.89-2.78)	1.79 (0.99-3.22)
	Television, radio, or newspaper (online)	2.41 (0.97-6.03)	2.52 (0.98-6.52)
	Other internet websites	1.28 (0.93-1.76)	1.24 (0.89-1.72)
	Social network services	1.61 (1.22-2.13)^f^	1.75 (1.31-2.35)^c^
	Instant messaging	1.67 (1.20-2.32)^f^	1.79 (1.27-2.51)^f^
**COVID-19 misinformation belief**			
	No	Reference	Reference
	Yes	7.38 (5.24-10.39)^c^	7.33 (5.17-10.38)^c^
**COVID-19 knowledge**			
	Low (0-24)	Reference	Reference
	High (25-35)	0.97 (0.74-1.26)	0.97 (0.74-1.27)
**COVID-19 preventive behaviors**			
	0-6 behaviors	Reference	Reference
	≥7 behaviors	1.08 (0.82-1.41)	1.13 (0.86-1.49)
**Anxiety symptom^h^**			
	No	Reference	Reference
	Yes	1.60 (1.12-2.29)^f^	1.80 (1.24-2.61)^f^
**Depressive symptom^i^**			
	No	Reference	Reference
	Yes	1.38 (1.03-1.84)^d^	1.47 (1.09-2.00)^d^
**Posttraumatic stress disorder symptom^j^**			
	No	Reference	Reference
	Yes	1.84 (1.34-2.53)^c^	1.97 (1.42-2.73)^c^

^a^All data were weighted by sex and age distribution of the general population in the Seoul metropolitan area in Korea.

^b^Adjusted for sex, age, highest education level, household arrangement, and monthly personal income.

^c^*P*<.001.

^d^*P*<.05.

^e^Average monthly income among employees was KRW 2,970,000 in 2018.

^f^*P*<.01.

^g^Multiple responses were allowed (the reference groups were those who responded “No”).

^h^Generalized Anxiety Disorder Questionnaire-2 (GAD-2) score ≥3.

^i^Patient Health Questionnaire-2 (PHQ-2) score ≥3.

^j^Primary Care Post-Traumatic Stress Disorder (PTSD) Screen for DSM-5 (PC-PTSD-5) score ≥3.

Subgroup analyses showed that the associations of misinformation exposure with misinformation belief, COVID-19 knowledge, preventive behaviors, and psychological distress differed according to the respondents’ demographics (sex, age, education, and monthly personal income; Multimedia Appendix 2). Among the respondents who reported misinformation exposure, misinformation belief was associated with lower COVID-19 knowledge levels (high: aOR 0.62, 95% CI 0.45-0.84 versus low) and fewer COVID-19 preventive behaviors (≥7 behaviors: aOR 0.54, 95% CI 0.39-0.74 versus 0-6 behaviors; Table 4).

**Table 4:** Associations of COVID-19 knowledge and number of COVID-19 preventive behaviors with COVID-19 misinformation belief among respondents who were exposed to misinformation (N=711)^a^.

Variables		Participants, n (%)	Misinformation belief (yes/no)	
			Crude odds ratio (95% CI)	Adjusted odds ratio (95% CI)^b^
**Knowledge**				
	Low (0-24)	259 (36.44)	Reference	Reference
	High (25-35)	452 (63.56)	0.59 (0.44-0.80)^c^	0.62 (0.45-0.84)^c^
**Preventive behaviors**				
	0-6 behaviors	305 (42.82)	Reference	Reference
	≥7 behaviors	407 (57.18)	0.51 (0.37-0.70)^d^	0.54 (0.39-0.74)^d^

^a^All data were weighted by sex and age distribution of the general population in the Seoul metropolitan area in Korea.

^b^Adjusted for sex, age, highest education level, household arrangement, and monthly personal income.

^c^*P*<.01.

^d^*P*<.001.

## Discussion

### Principal Findings

In this cross-sectional survey of Korean adults, more than two-thirds of the respondents reported COVID-19 misinformation exposure between the end of January 2020 and the end of April 2020, as COVID-19 evolved into a global pandemic. A previous study [28] identified a similar prevalence, where 70% of respondents reported misinformation exposure during the 2018 Ebola virus epidemic, affirming a substantial exposure risk to inaccurate or false health-related information during serious infectious disease outbreaks.

We identified that misinformation exposure was significantly associated with younger age, higher education levels, and lower incomes. Existing studies reported that younger people, including university students, preferred to obtain health information via online means and perceived themselves as having a high level of digital health literacy [29-31]. Such characteristics would expose young people to more COVID-19 misinformation and information. However, contrary to their perceptions, they lacked the skills to evaluate health resources and apply gathered information to health-related decisions [29-31]. This indicates that despite their proficiencies in using technology and the internet, effective interventions are required to improve young people’s digital health literacy, which is the ability to search for, understand, and critically evaluate health information through electronic sources, then apply gained knowledge to health issues [32]. Meanwhile, to our best knowledge, the associations between misinformation exposure and demographic characteristics have been underinvestigated in the existing literature. We performed subgroup analyses that offer additional details of interactions between the respondents’ demographic characteristics and COVID-19 variables. Further studies that provide in-depth understanding on how and why these demographic characteristics are associated with misinformation exposure will be useful.

Consistent with previous reports on the role of social media in health information dissemination and misinformation propagation [7,9,33,34], respondents in this study reported greater COVID-19 misinformation exposure through SNSs and instant messaging. This can also be attributed to the significance of social media like SNSs and instant messaging in Koreans’ daily lives, as those services also include product marketing, shopping, and payment services, contributing to Korea having the third highest social media usage penetration in the world [35].

Many recent studies have identified that the COVID-19 pandemic and fear of being infected have had negative effects on public mental health, reporting increased depression and anxiety [36-38]. The respondents in this study were similarly identified to be at high risk of psychological distress; compared to the nonexposure group, the misinformation exposure group notably had around 1.8 and 1.47 times higher anxiety and depressive symptoms, respectively. This demonstrates the alarming negative effect that misinformation can have on public mental health. Higher levels of social media use during the COVID-19 pandemic have been shown to result in higher levels of anxiety (OR 1.72) and a combination of depression and anxiety (OR 1.91) [39]. It is purported that prolonged and frequent use of social media throughout the ongoing pandemic increases exposure to misinformation along with accurate information. The mixture of accurate and false information can deliver conflicting messages and amplify uncertainties regarding COVID-19 and its perceived health risks [40], resulting in psychological distress [41,42]. A vicious cycle can be triggered, as evidence has shown that psychological distress itself can drive people to look for more information, which in turn causes further distress [43]. Despite the ongoing COVID-19 situation, this study also identified that the respondents had symptoms of PTSD in relation to the pandemic, raising concern that the psychological impact can persist and lead to poor physical health outcomes [44].

In this study, no association between misinformation exposure and COVID-19 knowledge as well as preventive behaviors was found. However, we identified that COVID-19 misinformation belief was negatively associated with COVID-19 knowledge and preventive behaviors, while it was positively associated with misinformation exposure. Similar to our findings, Allington et al [45] found that frequent use of SNSs, which propagate more misinformation than any other media [7], for COVID-19 information was associated with having conspiracy beliefs. Conspiracy beliefs, in turn, showed a negative relationship with COVID-19 preventive behaviors [45-47]. Vinck et al [28] also reported that the belief in Ebola virus misinformation resulted in a lower likelihood of adopting preventive behaviors.

The Health Belief Model (HBM) is a theoretical framework widely used in public health to understand heath behaviors for disease prevention (see Champion and Skinner [48]). It theorizes that people’s beliefs about their susceptibility to COVID-19 infection and its severity (collectively known as perceived threat), as well as perceptions about the benefits of and barriers to engaging in preventive behaviors will be predictive of their likelihood of engaging in those behaviors, while cues trigger engagement. Additionally, one’s understanding about COVID-19 can alter individual beliefs and thus indirectly influence behavior [48]. Based on the HBM, our findings suggest that those who believed in misinformation that they were exposed to had lesser accurate knowledge of COVID-19, which could include inaccurate knowledge about preventive behaviors. A less accurate understanding of COVID-19 in turn altered the perceptions they had about COVID-19, such as reduced perceived COVID-19 threat, reduced perceived benefits from preventive behaviors, heightened perceived benefits from inappropriate preventive behaviors, or heightened perceived barriers to preventive behaviors. These altered perceptions and beliefs regarding COVID-19 thus resulted in reduced engagement in recommended preventive behavior, as our findings show, or potentially increased engagement in inappropriate preventive behaviors. The HBM thus offers insights into interventions that can improve engagement in recommended preventive behaviors by delivering information targeted at the core HBM beliefs of perceived susceptibility, severity, benefits, and barriers [49]. Although our findings show no association between misinformation exposure and COVID-19 knowledge, increasing exposure to accurate or corrective COVID-19 information can be useful to denormalize misinformation beliefs, thus changing perceptions of COVID-19 to increase the likelihood of engaging in recommended preventive behaviors [4].

### Implications

As misinformation exposure is associated with misinformation belief, it is essential to manage and stem the propagation of misinformation through popular mediums like social media and counteract misinformation exposure with evidence-based information exposure. A study identified that official authorities had produced only a few COVID-19 information videos through a popular video streaming website (YouTube, a website with around 2 billion monthly users), while videos containing misinformation were disproportionately increasing [50]. Governments, health agencies, and researchers should take advantage of such social media outlets by producing and sharing evidence-based and corrective information through YouTube videos and simple but impactful infographics. Additionally, governments and health agencies can work closely with social media platforms to ensure that health-related information has increased visibility without involving unilateral censorship, develop misinformation alerts, and provide verification of information source quality, particularly in the event of global health crises [51]. These can accordingly mitigate the development of misinformation belief.

Increasing digital health literacy among the public, particularly young people, will also be essential, as misinformation exposure did not reflect improved COVID-19 knowledge or preventive behaviors but was associated with misinformation belief. Educational or training programs on digital health literacy should be developed and delivered to the public and can be introduced in schools to cultivate these skills from a young age.

In-depth, follow-up, and longitudinal studies that explore misinformation selection and beliefs, as well as how misinformation beliefs transform health behaviors will be beneficial as foundations to digital health literacy program development and anti-misinformation strategies.

### Limitations

This study has several limitations. First, the causal relationships between COVID-19 misinformation exposure and belief, COVID-19 knowledge, and psychological distress were uncertain due to the cross-sectional study design [52]. Second, we used survey questions to measure respondents’ COVID-19 misinformation exposure, COVID-19 knowledge, and preventive behaviors, which are not validated. Third, we collected self-reported data from the respondents that would cause recall and social desirability biases. Fourth, although all data were weighted according to the South Korea census data, there were relatively fewer people aged ≥60 years who participated in this study. We recruited adult respondents only and did not recruit teenagers (ie, those aged <20 years), who popularly use social media for information acquisition. The inclusion of younger people in future studies would provide additional evidence about their misinformation exposure and belief. Fifth, we conducted an online survey by recruiting panel members using a survey company and thus there is a possibility of sampling bias. For instance, those sampled were based in urban, not rural, areas and drawn from a high-income Asian country with prior experience of managing outbreaks of infectious diseases (eg, Middle East Respiratory Syndrome).

### Conclusion

We investigated the prevalence of misinformation exposure and factors that were associated with misinformation exposure and belief, including psychological distress, COVID-19 knowledge, and preventive behaviors in an adult population in the Seoul Metropolitan area, Korea. COVID-19 misinformation exposure was associated with misinformation belief, while misinformation belief was associated with poorer knowledge and engagement in fewer preventive behaviors. Given the potential of such misinformation to undermine global efforts in the COVID-19 response, public health strategies should be kept up-to-date and involve collaborations with multiple stakeholders, including social media platforms, to counter the proliferation of misinformation and win the fight against the infodemic.

## Appendix

Multimedia Appendix 1 Survey questionnaire.

Multimedia Appendix 2 Interaction effect of demographic characteristics with COVID-19 knowledge, preventive behaviours, misinformation belief, and psychological distress on misinformation exposure.

## Abbreviations

aOR: adjusted odds ratio
GAD-2: Generalized Anxiety Disorder-2
HBM: Health Belief Model
ICT: information and communication technology
OR: odds ratio
PHQ-4: Patient Health Questionnaire-4
PTSD: posttraumatic stress disorder
SNS: social networking service
WHO: World Health Organization
