# Supplementary material for: Associations Between COVID-19 Misinformation Exposure and Belief With COVID-19 Knowledge and Preventive Behaviors: Cross-Sectional Online Study
Source: J Med Internet Res. 2020 Nov 13;22(11):e22205. doi: 10.2196/22205 (PMC7669362; doi:10.2196/22205)
Supplement: Multimedia Appendix 1 [file jmir_v22i11e22205_app1.docx]

## Supplementary file 1. Survey questionnaire

## Part 1： Health information on COVID-19

1. In the past 3 months, did you encounter health information on the Coronavirus Disease 2019 (COVID-19) via the following sources? 【multiple answers allowed】

1 □ (Offline) TV/ radio/ newspaper/ magazine (excluding viewing or listening via the Internet)

2 □ (Online) TV/ radio/ newspaper/ magazine

3 □ Internet websites (apart from those in option 2)

4 □ Social networking services (e.g., Facebook, Instagram)

5 □ Instant messaging (e.g., WhatsApp, KakaoTalk)

6 □ Did not encounter any COVID-19-related information

1. In the past 3 months, did you encounter any of the following statements? 【multiple answers allowed】

1 □ Masks can be sterilised and reused after steaming with hot water

2 □ Masks can be reused after spraying alcohol on its surface

3 □ Flip the sides of a used mask to reuse it

4 □ Drinking alcohol/smoking can prevent COVID-19

5 □ Gargling can disinfect the respiratory tract and therefore prevent COVID-19

6 □ Drinking tea can prevent COVID-19

7 □ Taking antibiotics can prevent or treat COVID-19

8 □ A vaccine for the COVID-19 is available now

9 □ Only the elderly would become infected with the COVID-19

10 □ COVID-19 is artificially developed

11 □ Gargling with salt can prevent COVID-19

12 □ Basking in the sun can prevent COVID-19

13 □ None of the above

1. Which of the above information do you believe is correct?

1 □ None

2 □ Some are correct

3 □ Most are correct

4 □ All are correct

## Part 2：COVID-19 knowledge

1. What do you know about COVID-19【select one answer】

□ I do not know anything about it

□ A government’s scheme

□ A virus that can cause disease

□ A TV/ radio event

1. How does COVID-19 spread? 【multiple answers allowed】

1 □ Blood transfusions

2 □ Respiratory droplets from infected people

3 □ Airborne transmission

4 □ Direct contact with infected people

5 □ Touching contaminated objects/ surfaces

6 □ Unprotected sex

7 □ Contact with contaminated animals

8 □ Mosquito bites

9 □ Eating contaminated food

10 □ Drinking unpurified/untreated water

11 □ I do not know

1. What are the main symptoms of COVID-19? 【multiple answers allowed】

1 □ Fever

2 □ Cough

3 □ Shortness of breath and breathing difficulties

4 □ Muscle pain

5 □ Headache

6 □ Diarrhea

7 □ No symptoms

8 □ I do not know

1. Do you know how to prevent illness from COVID-19? 【multiple answers allowed】

1 □ Wear masks

2 □ Sleep under mosquito nets

3 □ Wash hands regularly using alcohol hand rub or soap and water

4 □ Drink only purified/treated water

5 □ Cover your mouth and nose when coughing or sneezing

6 □ Avoid close contact with anyone who has a fever and cough

7 □ Eliminate standing water

8 □ Fully cook meat and eggs

9 □ Avoid direct contact with live animals and uncleaned surfaces

10 □ Social distancing

11 □ I do not know

1. Do you think that COVID-19 induces fear? If yes, for whom?

1 □ Yes, for everyone

2 □ Yes, for most people

3 □ Yes, for some people

4 □ Yes, for a small minority

5 □ Yes, for very few people

6 □ No

## Part 3：COVID-19 preventive behaviours

1. What have you done to prevent getting ill with COVID-19 in the past 3 months?【multiple answers allowed】

1 □ Learnt how to wear a mask appropriately

2 □ Washed hands regularly using alcohol-based cleanser or soap and water

3 □ Covered mouth and nose when coughing or sneezing

4 □ Avoided close contact with anyone who had a fever and cough

5 □ Avoided going out

6 □ Stayed 1.5 meters away from others when going out

7 □ Avoided hand-shaking, hugging and kissing

8 □ Avoided social gatherings of more than 4 people

9 □ Avoided crowded places

10 □ Avoided high-risk places (e.g., bars)

## Part 4：Psychological health

1. In the past two weeks, how often have you experienced the following?【PHQ-4】

|  | None | Less than 7 days | 7 days or more | Almost every day |
| --- | --- | --- | --- | --- |
|  | 1 | 2 | 3 | 4 |
| 1. Feeling nervous, restless or irritable | □ | □ | □ | □ |
| 1. Unable to stop or control worrying | □ | □ | □ | □ |
| 1. Daily activities seem dull, or low motivation to do anything | □ | □ | □ | □ |
| 1. Low mood, depression or feelings of hopelessness | □ | □ | □ | □ |

1. In the past month, have you...【multiple answers allowed】【PC-PTSD-5】

1 □ had nightmares about the event(s) or thought about COVID-19 when you did not want to?

2 □ tried hard not to think about COVID-19 or went out of your way to avoid situations that reminded you of COVID-19?

3 □ been constantly on guard, watchful, or easily startled?

4 □ felt numb or detached from people, activities, or your surroundings?

5 □ felt guilty or been unable to stop blaming yourself or others for the current COVID-19 situation or any problems COVID-19 may have caused?

## Part 5：Demographics *mandatory fields

1. Gender:

1 □ Male

2 □ Female

1. Your age: ________
2. Marital status:

1 □ Never been married

2 □ Married/De facto partnership

3 □ Divorced/separated

4 □ Widowed

1. Your current living arrangement:

1 □ Living alone

2 □ Living with family

3 □ Living with others

1. Educational attainment:

1 □ Primary or below

2 □ Middle school

3 □ High school

4 □ Tertiary or above

1. Your current employment status:

1 □ Full-time work

2 □ Part-time work

3 □ Student

4 □ Housekeeper

5 □ Retiree

6 □ Unemployed

1. Your monthly personal income (average)

1 □ KRW 1,000,000 or less

2 □ KRW 1,000,001 - 2,000,000

3 □ KRW 2,000,001 - 3,000,000

4 □ KRW 3,000,001 - 4,000,000

5 □ KRW 4,000,001 - 5,000,000

6 □ KRW 5,000,001 or more

~End of questionnaire~
