# Supplementary material for: Associations Between COVID-19 Misinformation Exposure and Belief With COVID-19 Knowledge and Preventive Behaviors: Cross-Sectional Online Study
Source: J Med Internet Res. 2020 Nov 13;22(11):e22205. doi: 10.2196/22205 (PMC7669362; doi:10.2196/22205)
Supplement: Multimedia Appendix 2 [file jmir_v22i11e22205_app2.docx]

**Supplementary file 2. Interaction effect of demographic characteristics with COVID-19 knowledge, preventive behaviours, misinformation belief and psychological distress on misinformation exposure**

|  | |  | **Adjusted ^a^ Odds Ratio (OR) 95% CI** | | **P for interaction** |
| --- | --- | --- | --- | --- | --- |
|  |  | | **Sex** | |  |
| **COVID-19** |  | | **Male** | **Female** |  |
| Misinformation belief | No | | REF | REF | < 0.001 |
|  | Yes | | 11.54 (6.76-19.69)^***^ | 4.94 (3.10-7.90)^***^ |  |
| Knowledge | Low (0-24) | | REF | REF | 0.669 |
|  | High (25-35) | | 0.90 (0.61-1.31) | 1.10 (0.75-1.61) |  |
| Preventive behaviors | 0 - 6 behaviors | | REF | REF | 0.573 |
|  | 7 or more behaviors | | 1.29 (0.88-1.91) | 1.04 (0.70-1.55) |  |
| Anxiety symptom ^b^ | No | | REF | REF | 0.016 |
|  | Yes | | 2.22 (1.30-3.79)^**^ | 1.61 (0.95-2.73) |  |
| Depressive symptom ^c^ | No | | REF | REF | 0.073 |
|  | Yes | | 1.52 (0.98-2.38) | 1.53 (1.00-2.36) |  |
| PTSD symptom ^d^ | No | | REF | REF | 0.006 |
|  | Yes | | 1.33 (0.75-2.34) | 3.28 (1.65-6.53)^**^ |  |
|  |  | | **Age** | |  |
|  |  | | **20-59** | **60-69** |  |
| Misinformation belief | No | | REF | REF | < 0.001 |
|  | Yes | | 6.60 (4.56-9.57)^***^ | 11.43 (4.01-32.57)^***^ |  |
| Knowledge | Low (0-24) | | REF | REF | 0.003 |
|  | High (25-35) | | 1.02 (0.76-1.37) | 0.88 (0.43-1.78) |  |
| Preventive behaviors | 0 - 6 behaviors | | REF | REF | 0.002 |
|  | 7 or more behaviors | | 1.11 (0.82-1.50) | 2.15 (0.98-0.47) |  |
| Anxiety symptom ^b^ | No | | REF | REF | < 0.001 |
|  | Yes | | 1.83 (1.19-2.81)^**^ | 1.34 (0.58-3.12) |  |
| Depressive symptom ^c^ | No | | REF | REF | < 0.001 |
|  | Yes | | 1.31 (0..94-1.84) | 1.82 (0.84-3.94) |  |
| PTSD symptom ^d^ | No | | REF | REF | < 0.001 |
|  | Yes | | 1.74 (1.09-2.76)^*^ | 6.01 (1.57-23.04)^**^ |  |
|  |  | | **Education** | |  |
|  |  | | **≤ High school** | **≥ Tertiary** |  |
| Misinformation belief | No | | REF | REF | < 0.001 |
|  | Yes | | 2.45 (1.32-4.55)^**^ | 11.59 (7.39-18.17)^***^ |  |
| Knowledge | Low (0-24) | | REF | REF | 0.148 |
|  | High (25-35) | | 1.14 (0.65-1.99) | 0.89 (0.65-1.22) |  |
| Preventive behaviors | 0 - 6 behaviors | | REF | REF | 0.098 |
|  | 7 or more behaviors | | 1.65 (0.92-2.94) | 1.04 (0.75-1.42) |  |
| Anxiety symptom ^b^ | No | | REF | REF | 0.002 |
|  | Yes | | 1.10 (0.53-2.27) | 2.02 (1.29-3.18)^**^ |  |
| Depressive symptom ^c^ | No | | REF | REF | 0.003 |
|  | Yes | | 2.46 (1.30-4.64)^**^ | 1.17 (0.82-1.68) |  |
| PTSD symptom ^d^ | No | | REF | REF | 0.004 |
|  | Yes | | 2.53 (1.08-5.93)^*^ | 1.88 (1.14-3.10)^*^ |  |
|  |  | | **Monthly personal income** ^e^ | |  |
|  |  | | **< 3,000,000** | **≥ 3,000,000** |  |
| Misinformation belief | No | | REF | REF | < 0.001 |
|  | Yes | | 4.77 (3.05-7.46)^***^ | 13.29 (7.47-23.63)^***^ |  |
| Knowledge | Low (0-24) | | REF | REF | 0.080 |
|  | High (25-35) | | 1.10 (0.76-1.59) | 0.85 (0.58-1.25) |  |
| Preventive behaviors | 0 - 6 behaviors | | REF | REF | 0.027 |
|  | 7 or more behaviors | | 1.41 (0.97-2.06) | 0.88 (0.58-1.31) |  |
| Anxiety symptom ^b^ | No | | REF | REF | < 0.001 |
|  | Yes | | 1.21 (0.74-1.98) | 2.69 (1.55-4.70)^***^ |  |
| Depressive symptom ^c^ | No | | REF | REF | 0.006 |
|  | Yes | | 1.32 (0.87-2.00) | 1.63 (1.04-2.55)^**^ |  |
| PTSD symptom ^d^ | No | | REF | REF | 0.001 |
|  | Yes | | 1.56 (0.87-2.81) | 2.32 (1.26-4.27)^**^ |  |

Note: All data were weighted by sex and age distribution of the general population in the Seoul metropolitan area in Korea; ^a^ Adjusted for sex, age, highest education level, household arrangement, and monthly personal income; ^b^ Generalized Anxiety Disorder Questionnaire-2 (GAD-2) score ≥ 3; ^c^ Patient Health Questionnaire-2 (PHQ-2) score ≥ 3; ^d^ Primary Care Post-Traumatic Stress Disorder (PTSD) Screen for DSM-5 (PC-PTSD-5) score ≥ 3; ^e^ US$1 = KRW1,200; ^*^ *P* < 0.05, ^**^*P* < 0.01, ^***^*P* < 0.001.
